# Alternative mRNA Editing in Trypanosomes Is Extensive and May Contribute to Mitochondrial Protein Diversity

**DOI:** 10.1371/journal.pone.0001566

**Published:** 2008-02-13

**Authors:** Torsten Ochsenreiter, Michael Cipriano, Stephen L. Hajduk

**Affiliations:** 1 Department of Biochemistry and Molecular Biology, University of Georgia, Athens, Georgia, United States of America; 2 Department of Medical Microbiology, University of California Davis, Davis, California, United States of America; Dalhousie University, Canada

## Abstract

The editing of trypanosome mitochondrial mRNAs produces transcripts necessary for mitochondrial functions including electron transport and oxidative phosphorylation. Precursor-mRNAs are often extensively edited by specific uridine insertion or deletion that is directed by small guide RNAs (gRNAs). Recently, it has been shown that cytochrome c oxidase subunit III (COXIII) mRNAs can be alternatively edited to encode a novel mitochondrial membrane protein composed of a unique hydrophilic N-terminal sequence of unknown function and the C-terminal hydrophobic segment of COXIII. To extend the analysis of alternative editing in *Trypanosoma brucei* we have constructed libraries with over 1100 full-length mitochondrial cDNAs and the sequences of over 1200 gRNA genes. Using this data, we show that alternative editing of COXIII, ATPase subunit 6 (A6), and NADH dehydrogenase subunits 7, 8 and 9 (ND7, 8, 9) mRNAs can produce novel open reading frames (ORFs). Several gRNAs potentially responsible for the alternative editing of these mRNAs were also identified. These findings show that alternative editing of mitochondrial mRNAs is common in *T. brucei* and expands the diversity of mitochondrial proteins in these organisms.

## Introduction

RNA editing in trypanosome mitochondria is a posttranscriptional process of endonuclease cleavage, uridine insertion or deletion and ligation of mRNAs that is directed by small non-coding guide RNAs (gRNAs) (reviewed in [1]–[3]). Functionally, RNA editing has been shown to correct for frameshift mutations, form initiation codons and create entire ORFs [4]–[6]. The genes encoding the trypanosome mitochondrial mRNAs are found on maxicircles, while gRNAs are largely encoded by genes located on minicircles. These two classes of mitochondrial DNA form a unique structure called the kinetoplast composed of thousands of minicircles and approximately 50 maxicircles topologically interlocked to form a huge network structure. Uridine insertion/deletion editing effectively combines information at the RNA level that is encoded separately, on minicircles and maxicircles in the mitochondrial genome of trypanosomes [7]. The complete and correct editing of trypanosome mitochondrial mRNAs is essential to the organism and is critical for the production of conventional mitochondrial proteins including subunits of the mitochondrial respiratory chain and the ATP synthase. Initial sequencing studies of extensively edited COXIII, ND7 and A6 mRNAs from *T. brucei* showed that the repertoire of distinctly edited mRNAs from a single mitochondrial gene was extensive but this sequence diversity was largely dismissed as a reflection of the inaccuracy and inefficiency of RNA editing resulting in incomplete or incorrectly edited RNAs that were functionally unimportant [8]–[10]. We have recently identified an alternatively edited COXIII mRNA and have shown its protein product associates in a high molecular weight complex in trypanosome mitochondrial membranes [11]. The detection of an alternatively edited mRNA, and its protein product, suggested that differential editing of pre-mRNAs could play a role in expanding protein diversity by the production of functionally discrete isoforms of conventional mitochondrial proteins with different enzymatic activities, substrate specificities, subcellular localization or altered abilities to interact with other proteins, DNA or RNAs. Alternatively edited mRNAs might also create proteins with novel functions needed for the peculiar biogenesis and regulation of the mitochondrion of trypanosomes [12], [13].

Mitochondrial biogenesis in trypanosomes is developmentally regulated. In the bloodstream form of the parasite many of the mitochondrial respiratory complex proteins including cytochrome c oxidase and cytochrome c reductase complexes are down regulated. This is in good agreement with the physiological data that shows the absence of any oxidative phosphorylation in this developmental stage [14]. The transcription and editing of some respiratory complex genes, including COXIII, however is not down regulated in the bloodstream stage. This suggests that the edited mRNAs from these genes may encode proteins that are functionally distinct from conventional respiratory complex proteins. Similarly, mRNAs from the NADH dehydrogenase (respiratory Complex I) gene ND7 are differentially edited during the developmental cycle of *T. brucei* [6]. In this case, editing of ND7 mRNA in the insect vector stage of the parasite occurs only in its 5′ domain leaving the 3′ domain pre-edited. These studies did not investigate the presence of protein products from the alternative ND7 mRNAs but suggested that differential editing of ND7 mRNA might produce isoforms of ND7 specific to the developmental stages of the trypanosome.

We initiated an analysis of the extent of alternative mRNA editing by large scale sequencing of full-length cDNAs from the bloodstream developmental stage of *T. brucei* [11], [15]. The diversity of edited mitochondrial mRNAs suggested the formation of alternative ORFs might be widespread in trypanosomes. Here we show that mRNAs from five *T. brucei* mitochondrial genes are alternatively edited giving rise to minor amino acid substitutions, extended ORFs or extensive sequence changes when compared with pre-edited or *bona fide* edited transcripts. In addition, several gRNAs responsible for the alternative editing of these mRNAs are identified. Based on these data we propose that alternative editing of mitochondrial mRNAs is widespread in *T. brucei* and expands mitochondrial protein diversity.

## Results

### Alternative Editing of ND7, ND8 and ND9 mRNAs

In the insect developmental stage of *T. brucei* the mitochondrial NADH dehydrogenase (Complex I) functions as an electron acceptor for NADH thereby facilitating electron transport and playing a key role in establishment of mitochondrial membrane potential and oxidative phosphorylation [16]. Bloodstream *T. brucei* lacks detectable cytochrome c reductase (Complex III) and cytochrome c oxidase (Complex IV) activity. Instead Complex I donates a pair of electrons to a plant-like alternative terminal oxidase completing the reoxidation of NADH formed glycolytically during substrate level phosphorylation [17].

**Figure 1:**
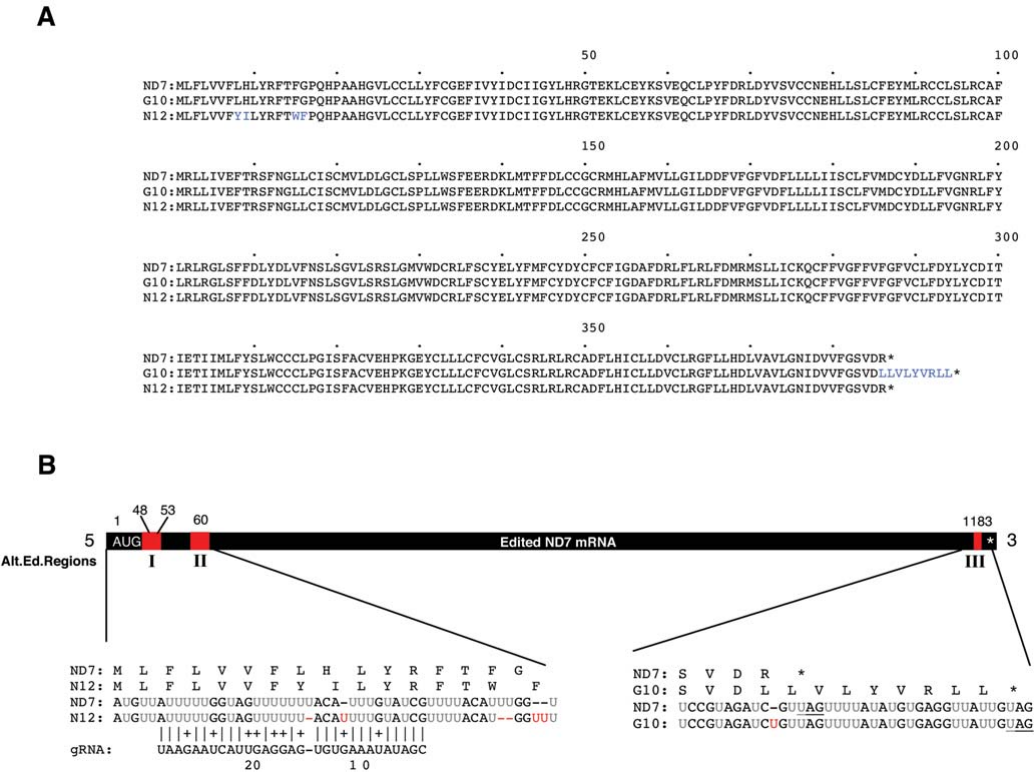
Alternative editing of ND7 mRNAs. A) Alignment of the predicted amino acid sequence of ND7 and the alternatively edited ND7 cDNAs ND7-G10 and ND7-N12. Amino acids changed by alternative editing are in blue. B) Bar depicts the alternatively edited mRNA sequences ND7-G10 and ND7-N12. Alignment of the RNA sequences for ND7, ND7-N12 and ND7-G10 from the alternatively edited regions I, II and III are shown below the bar. In black are the pre-edited residues, in grey the *bona fide* edited residues and in red are the alternatively edited residues from regions I–III. Above the RNA sequences are the corresponding amino acid sequences. A predicted gRNA for N12 shows perfect complementarity (allowing for G:U) to ND7-N12 over 30 base pairs while having mismatches to the ND7 sequence at nucleotide positions 10 and 14 of the gRNA. Vertical bars indicate A:U or G:C base pairing; crosses indicates G:U base pairing. Underlined sequence shows UAG termination codon. Star depicts amber codon in the amino acid sequence. Red Us indicate alternatively inserted/not inserted Us when compared with the ND7 sequence.

To determine whether alternative mRNA editing could produce isoforms of the Complex I subunits we carried out a detailed analysis of cDNA sequence data from extensively edited ND7, ND8 and ND9 mRNAs isolated from the bloodstream developmental stage of *T. brucei*. The sequence of ND7 mRNA was originally deduced based on the consensus sequence of 77 short cDNAs and direct RNA sequencing [6]. The *bona fide* ND7 mRNA contained 551 uridines added and 86 uridines deleted by RNA editing to form the 1246 nts mRNA. We have extended the characterization of ND7 RNA editing by detailed analysis of 21 full-length cDNAs and examination of the protein coding potential for each transcript. The sequence of ND7 mRNAs revealed two alternatively edited RNAs with unique ORFs (Figure 1). It was previously noted that differential editing of ND7 mRNAs could lead to diverse ORFs and dramatic differences in editing correlated with the developmental stage of the parasite [6]. We have verified similar changes leading to frame shifting and amino acid substitutions in the ND7 mRNAs in bloodstream *T. brucei*.

The complete cDNA sequence of ND7 mRNA produces a predicted protein coding sequence of 386 amino acids. In addition to this fully edited ND7 sequence, we identified two alternatively edited ND7 mRNAs containing a total of seven alternatively inserted uridines at five editing sites (Figure 1A). One alternatively edited mRNA, designated ND7-G10, differs from the fully edited ND7 mRNA by the addition of a single uridine at nucleotide 1183 resulting in a frameshift that extends the predicted coding sequence of this alternatively edited mRNA by 21 nucleotides to a UAG termination codon. The C-terminus of this predicted ND7 isoform is rich in hydrophobic amino acids (two valine and five leucine residues) suggesting that it may uniquely associate with the mitochondrial membrane or assemble differently in the NADH dehydrogenase complex. Another differentially edited ND7 mRNA, designated ND7-N12, differs from the fully edited ND7 mRNA at four sites in two regions of the mRNA near the translation initiation codon resulting in four non-conservative amino acid substitutions (Figure 1A, B). Both alternatively edited transcripts use the *bona fide* ND7 initiation codon (AUG). In our gRNA gene database, we identified a gRNA gene complementary to 30 nts of the ND7-N12 cDNA that can direct the alternative editing of nucleotides 23–26 in region I. The gRNA directing the alternative editing of the ND7-G10 mRNA has not been identified.

**Figure 2:**
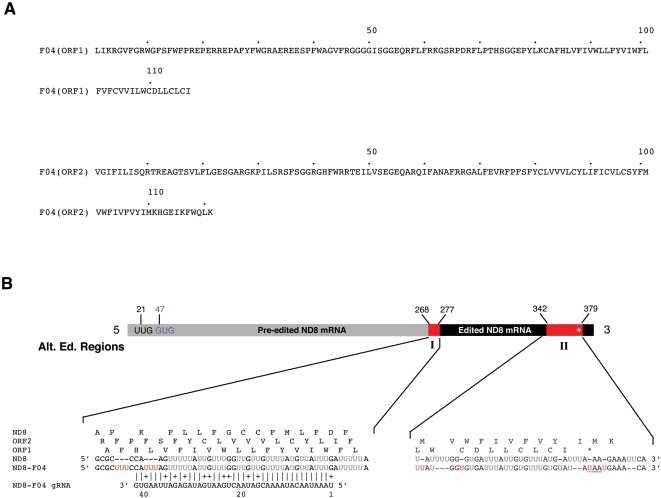
Alternative editing of ND8 mRNAs. A) Protein sequence of ORF1 and ORF2, both sequences show no similarity to the *bona fide* ND8 (not shown). B) Bar depicts the mRNA sequence ND8-F04. Alignment of the RNA sequences for ND8, and ND8-F04 from the alternatively edited regions I, and II are shown below the bar. In black are the pre-edited residues, in grey the *bona fide* edited residues and in red are the alternatively edited residues from regions I–II. In black is the pre-edited, in grey the *bona fide* edited region and in red are the alternatively edited regions I and II. Alternative start codons UUG and GUG are shown in black and purple, respectively. Alignment of the RNA sequence of ND8 and cDNA ND8-F04 from the alternatively edited region I and II (red). Depicted above the RNA sequences are the corresponding amino acid sequences. Predicted gRNA for region I showing perfect complementarity (allowing for G:U) to ND8-F04 over 41 base pairs while having three mismatches to the ND8 sequence at positions 38 to 40 of the gRNA. Vertical bars indicate A:U or G:C base pairing; crosses indicates G:U base pairing. Red Us indicate alternatively inserted Us when compared with the *bona fide* ND8 sequence. Star depicts termination codon in the amino acid sequence. Underlined sequence depicts stop codon in the nucleotide sequence.

Analysis of 78 full-length cDNAs from the ND8 gene also revealed alternatively edited mRNA. The ND8-F04 cDNA and two other identical sequences contained two regions where a total of 11 uridines are inserted alternatively at seven editing sites (Figure 2A, Table 1). This transcript contained two ORFs of similar size (Figure 2A). ORF1 started with an UUG (Leu) initiation codon 21 nucleotides downstream of the predicted 5′end and terminated with a UAA termination codon produced by alternative editing after 117 amino acids. When compared to the public databases this sequence showed no similarity to any known sequence. The second ORF (ORF2) started 47 nucleotides downstream of the 5′ end with a GUG (Val) start codon and had no termination codon in the ND8 coding sequence, however a termination codon (UAA) was found in the poly A tail which terminated the sequence after 121 amino acids. The predicted amino acid sequence (ORF2) showed weak similarity over a short stretch to a hypothetical protein from *Plasmodium chabaudi* (XP_744827).

**Table 1:** Features of alternatively edited transcripts

	ND7	ND8	ND9	COXIII	A6
**# Sequences/fully edited**	21/4	78/11	126/18	223/4	69/7
**# Alt. sequences**	1×G10, 1×N12	4×F04	6×F12	2×K12*	3×D08
**Alt. editing sites**	5	7	67	17	5
**Alt. ORFs**	2	2	1	1	1
**Alt. start codon**	Only AUG	UUG (ORF1)	GUG/UUG	UUG	UUG
		GUG (ORF2)			
**Alt. stop codon/site**	UAG 21nt downstream of *bona fide* UAG	UAA 34nt downstream *of bona fide* UAG	Uses the *bona fide* UAA	UAG 28nt downstream of *bona fide* UAA	Uses the *bona fide* UAG
**Alt. gRNAs needed/identified**	2/1	2/1	5/1	4/1	3/1
**Type of alternative editing**	AChange	TChange	ORFCreation	AChange	UTRChange
	TChange	XCreation		TChange	XCreation
				UTRChange	
				XCreation	

AChange, change in amino acid composition; TChange, change of termination codon; XCreation, creation of a junction sequence; UTRChange, change of UTR sequence; ORFCreation, creation of entire open reading frames by alternative editing.

* we have identified 31 cDNA sequences that form alternative open reading frames similar to K12 with minor amino acid changes in the junction region, these sequences are not identical to K12.

Screening our gRNA database, we identified a corresponding gRNA gene that would be able to guide for the insertion of the 3′ most alternatively inserted uridine residues at nucleotide position 274-76 (Figure 2B) [15]. The gRNA gene matched with the alternative transcript over 41 bp with 11 G:U base pairings. The five 3′ most nucleotides of the gRNA gene guided for the alternative insertion pattern observed in the ND8 transcript.

**Figure 3:**
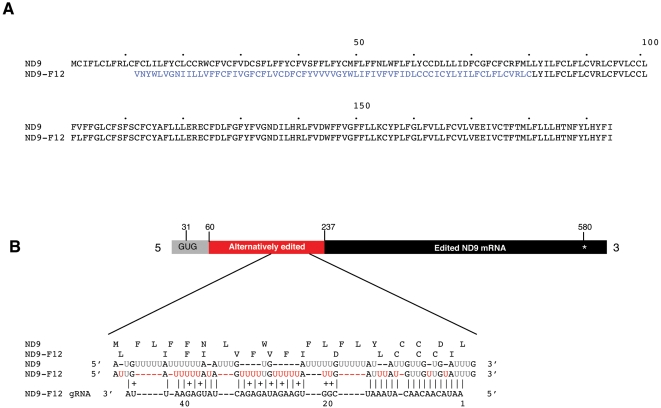
Alternative editing of ND9 mRNA. A) Alignment of the predicted amino acid sequence of ND9 and the alternatively edited cDNA ND9-F12. Amino acids changed by alternative editing are in blue. B) Bar depicts the ND9-F12 mRNA. Alignment of the RNA sequences for ND9, and ND9-F12 from the alternatively edited region are shown below the bar. In black are the pre-edited residues, in grey the *bona fide* edited residues and in red are the alternatively edited residues. The initiation codon GUG is shown in black on the bar. Above the RNA sequences are the corresponding amino acid sequences. Predicted gRNA for ND9-F12 shows perfect complementarity (allowing for G:U) to F12 over 44 base pairs while having 14 mismatches to the ND9 sequence in the best possible alignment (not shown). Vertical bars indicate A:U or G:C base pairing; crosses indicates G:U base pairing. Star indicates a termination codon. Red Us indicate alternatively inserted Us when compared with the ND9 sequence.

Analysis of 126 full-length ND9 cDNAs identified six alternatively edited transcripts of identical sequence, containing a novel open reading frame (designated ND9-F12, Figure 3A). The mRNA sequence was identical to the consensus for ND9 in the 3′ terminal 366 nucleotides while an extended 5′ region of the pre-mRNA was edited differently at 67 editing sites. An alternative ORF was identified to start with GUG (Val) at nucleotide 31 and terminated at nucleotide 580 with the *bona fide* termination codon of ND9 mRNA (UAA). The C-terminal 113 amino acids of the predicted alternative protein were identical to ND9, whereas the N-terminal sequence of 67 amino acids showed no homology to ND9. When this sequence was compared to the public databases no sequences with significant similarity were found.

We identified a gRNA gene, as well as the expressed gRNA, in our cDNA libraries predicted to direct the alternative insertion of at least 23 uridine residues (Figure 3B) assuming an 8 bp gRNA anchor length. The gRNA showed perfect complementarity over 44 nucleotides with the alternative transcript and no significant complementarity to the fully edited ND9 transcript.

### Alternative Editing of COXIII mRNA

**Figure 4:**
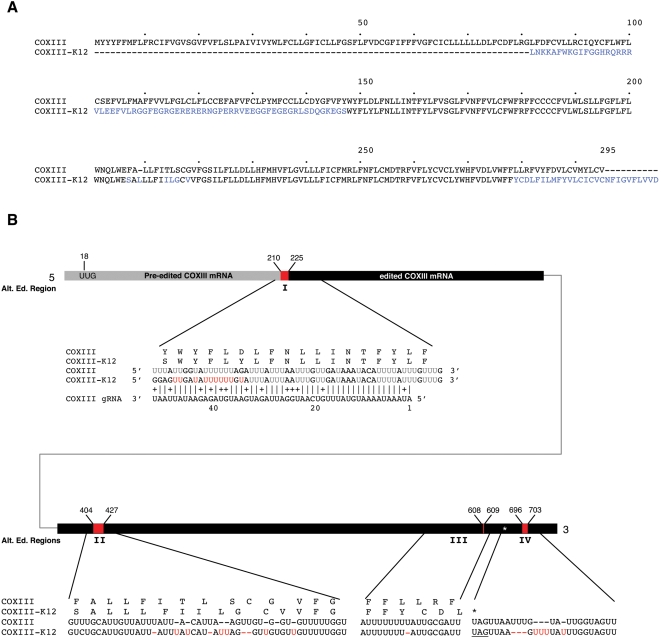
Alternative editing of COXIII mRNA. A) Alignment of the predicted amino acid sequence of COXIII and the alternatively edited COXIII-K12 cDNA. B) Bar represents the mRNA sequence of COXIII-K12. 5′ and 3′ region are connected by a thin line. Alignment of the RNA sequences for COIII, and COIII-K12 from the alternatively edited regions I, II, III and IV are shown below the bar. In black are the pre-edited residues, in grey the *bona fide* edited residues and in red are the alternatively edited residues from regions I–IV. The UUG initiation codon is shown in black. Alignment of the RNA sequence of COXIII and cDNA K12 from the alternatively edited region I–IV (red). Above the RNA sequences are the corresponding amino acid sequences of K12 and COXIII. Predicted gRNA sequence shows perfect complementarity (allowing for G:U) to K12 over 51 base pairs while having one mismatch to the COXIII sequence at position 38 of the gRNA. Vertical bars indicate A:U or G:C base pairing; crosses indicates G:U base pairing. Red Us indicate alternatively inserted Us when compared with the COXIII sequence. Star depicts termination codon in the amino acid sequence. Underlined sequence depicts stop codon in the nucleotide sequence.

We previously reported the alternative editing of COXIII mRNA and have identified a protein, AEP-1, encoded by this mRNA [11]. Another alternatively edited COXIII mRNA, COXIII-K12, shows a similar sequence pattern to AEP-1 mRNA containing a 210 nt pre-edited 5′ region with an ORF joined at an alternatively edited junction region (nucleotides 210 to 225) to a sequence identical to COXIII resulting in an ORF of 223 amino acids (Figure 4A, B; Table 1). Editing at the junction region of COXIII-K12 results in a +1 frameshift to create an extended ORF with the 5′ pre-edited and 3′ edited ORFs joined. In addition, changes in the position of uridine insertions result in three amino acid substitutions in the junction region.

We identified a gRNA gene for the alternative editing site of region I, between nucleotides 210 and 225, of the COXIII-K12 cDNA. The gRNA showed perfect complementarity over 50 nucleotides with the alternative COXIII-K12 and is predicted to direct the insertion of 10 uridines unique to the COXIII-K12 cDNA (Figure 4B).

In addition to alternative editing of the COXIII-K12 mRNA at the junction of the pre-edited and edited COXIII mRNA, three other alternatively edited regions were identified in this cDNA (Figure 4B). The alternatively edited region II (nucleotides 404 to 427) differs from the consensus COXIII sequence in the number of uridines added at nine sites resulting in eight predicted changes in the amino acid sequence of COXIII-K12. Alternative editing of region III (nucleotides 608 to 609) results in the addition of one less uridine thereby shifting the predicted reading frame of the COXIII-K12. The shift in reading frame results in the addition of 10 amino acids until the in-frame stop codon (UAG) at nucleotide 690. Finally, a fourth alternative editing site for the COXIII-K12 mRNA was observed in the 3′ untranslated region (UTR) starting 11 nucleotides downstream of the UAG termination codon. We have not identified gRNAs or gRNA genes that could direct the alternative editing at the sites II, III and IV of the COIII-K12 mRNA.

### Alternative Editing of A6 mRNA

**Figure 5:**
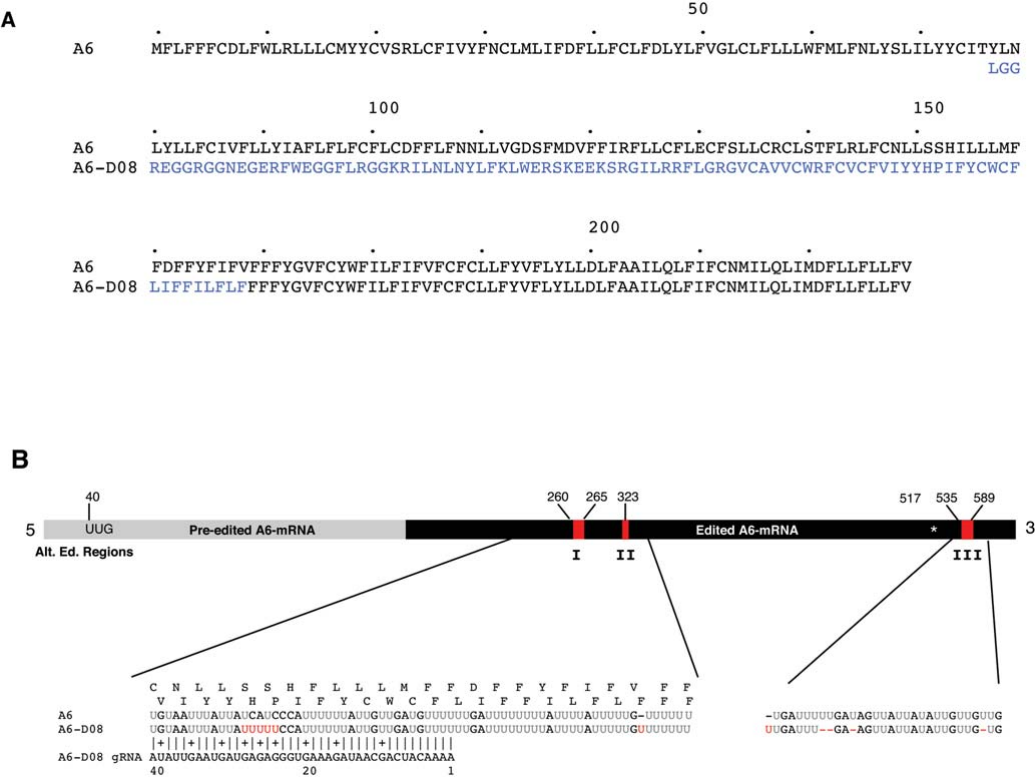
Alternative editing of A6 mRNA. A) Alignment of the predicted amino acid sequence of A6 and the alternatively edited cDNA A6-D08. B) Alignment of the RNA sequences for A6, and A6-D08 from the alternatively edited regions I, and II are shown below the bar. In black are the pre-edited residues, in grey the *bona fide* edited residues and in red are the alternatively edited residues from regions I–III.Bar represents the mRNA sequence of A6-D08. The UUG initiation codon is shown in black. Alignment of the RNA sequence of A6 and A6-D08 from the alternatively edited regions I, II and III (red). Depicted above the RNA sequences are the corresponding amino acid sequences of D08 and A6. The predicted gRNA shows perfect complementarity (allowing for G:U) to A6-D08 over 40 base pairs while having two mismatches to the A6 sequence at position 26-27 of the gRNA. Vertical bars indicate A:U or G:C base pairing; crosses indicates G:U base pairing. Red Us indicate alternatively inserted Us when compared with the A6 sequence. Star shows termination codon in the amino acid sequence.

Analysis of 69 full length A6 cDNAs also identified three alternatively edited cDNAs of identical sequence, designated A6-D08. This sequence contained an ORF that differed from the A6 consensus sequence (Figure 5A). The ORF was initiated by an alternative UUG start codon at nucleotide position 40 and terminated by the *bona fide* A6 stop codon. The mRNA sequence of A6-D08 was identical to the consensus for A6 at nucleotides 323 to 517. The 5′ 208 nucleotides were the pre-edited sequence encoded by the A6 gene while three regions (I, 260–265; II, 323 and III 517–535 Table 1) were edited to a unique sequence. The formation of the A6-D08 ORF requires the correct alignment of three distinct ORFs encoded by pre-edited, alternatively edited and fully edited A6 at two junctions, one between the pre-edited and edited sequence and the second at the alternatively edited region II (Figure 5B). When the corresponding amino acid sequence was compared to the public databases no sequences with significant similarity were found. We have identified a gRNA gene to the alternatively edited region I of A6-D08 mRNA. The gRNA is predicted to have perfect complementarity over 40 bp to the alternative A6-D08 mRNA from nucleotide position 248-288.

## Discussion

The discovery of RNA editing provided an explanation for the seeming lack of several typical mitochondrial genes in trypanosomes [4]. Many mitochondrial mRNAs are so extensively edited that their gene sequences failed to reveal the ORFs for conventional protein products. The insertion and deletion of hundreds of uridines leads to the formation of functional mRNAs for mitochondrial proteins including components of the major mitochondrial respiratory complexes and the ATP synthase. These findings led to the widely accepted conclusion that the function of RNA editing was to amend the incomplete coding information of trypanosome mitochondrial genes to produce mRNA for conventional mitochondrial proteins.

The recent discovery that alternative RNA editing of a COXIII mRNA can create an mRNA that is translated to produce a novel protein, AEP-1, suggests that alternative editing may provide a powerful means to fine-tune and diversify trypanosome mitochondrial gene products [11]. In this paper, we have extended our analysis of the diversity of *T. brucei* mitochondrial mRNAs and report the sequence of alternatively edited A6, ND7, ND8, ND9 mRNAs and a second alternatively edited mRNA for COXIII.

Based on the widespread occurrence of alternatively edited mRNAs shown in this study, we propose that alternative RNA editing in trypanosomes be defined as any uridine addition or deletion that produces ORFs which differ from the anticipated coding sequence for mitochondrial proteins. We show that alternative mRNA editing can affect protein-coding sequences in five ways. 1) Alternative uridine insertion or deletion can cause amino acid substitutions within coding sequences (ND7-N12, COXIII-K12). 2) Alternative editing can occur at pre-edited/edited junctions to create extended ORFs composed of 5′ pre-edited and 3′ edited sequences (ND8-F04, COXIII-K12, A6-D08). 3) Alternative uridine insertion and deletion in mRNAs can form new or eliminate canonical translation termination codons to extend or shorten ORFs (ND7-N12 and COXIII-K12), in some instances this occurs in the poly(A) tail (A6,-D08) as has been described previously [5], [18]. 4) Alternative editing of an mRNA can be extensive resulting in formation of RNA sequences containing unique ORFs differing from those in either the pre-edited or fully edited mRNA (A6, ND9). 5) Alternative editing can change the UTR sequence and thereby might affect regulation of expression or stability of certain transcripts (COXIII-K12, A6-D08).

Currently, the function of alternative mRNA editing in trypanosome mitochondria is unknown but by analogy to alternative RNA splicing in many eukaryotes it seems likely that a major function for alternative editing will be to generate isoforms of a protein with different enzymatic activities, substrate specificities, cellular localization and altered ability to interact with other proteins or nucleic acids [19]–[21]. Alternative editing may also be important in producing mitochondrial proteins with novel functions specific to trypanosomes [7]. Consistent with this possibility, AEP-1 is encoded by an alternately edited COXIII mRNA that is expressed in the bloodstream developmental stage of *T. brucei* when the expression of all other nuclear and mitochondrial encoded subunits of the cytochrome c oxidase is suppressed and the activity of the complex is not detectable. The absence of cytochrome c oxidase activity suggests that AEP-1 has a novel function in trypanosome mitochondria and supports the idea that alternative mRNA editing is likely to provide a mechanism for creating mitochondrial protein diversity. This is supported by previous studies in Kinetoplastidae that showed that RNA editing could be a source of genetic variation [18]. These studies provided evidence that the predicted proteins of extensively edited genes accumulate mutations at a higher frequency than their unedited homologues in closely related species. Landweber and Gilbert concluded that RNA editing is a surprisingly inefficient mechanism for conserving amino-acid sequences.

With the data presented here we believe the rapid evolution of proteins from extensively edited transcripts is a consequence of RNA editing being a diversifying, rather than a conservation/repair mechanism.

The production of variations of the limited mitochondrial encoded proteins may be particularly important in trypanosomes as they use diverse mechanisms for the maintenance of their mitochondrial genome, and the developmental regulation of mitochondrial biogenesis and energy production. Furthermore, a correlation in the developmental regulation of mitochondrial biogenesis and both the extent and the diversity of RNA editing has been observed in *T. brucei* [22]–[25].

One of the underlying premises of RNA editing has been that ORFs were formed in the mRNA by uridine addition or deletion thus, pre-edited mRNA sequences could be considered the equivalent to non-coding RNA and would be anticipated to lack any significant ORFs. In our analysis of extensively edited mRNA, however we noticed that the pre-edited mRNAs contained multiple, extended ORFs (Table S1), while transcripts that are never edited, for example ND5, contain only one ORF.

So why do the extensively edited trypanosome mitochondrial genes contain multiple ORFs although their mRNAs do not contain complete coding information until edited? The importance of ORFs in pre-edited mRNA becomes clear if we consider it in conjunction with alternative editing. We have shown that ORFs in the pre-edited mRNA are required for the creation of the alternative ORFs for ND8, COXIII and A6 (Figures 2, 4 and 5). In each of these alternatively edited mRNAs the pre-edited mRNA sequence contains the initiation codon as well as 5′ coding sequence, which is joined to a second ORF by alternative RNA editing. Together the pre-edited reading frame and the edited or alternatively edited reading frame create an extended, novel coding sequence. Seven of the nine pan edited genes contain up to three ORFs that start within 60 nucleotides of the 5′ end of the pre-edited transcript (Table S1). This increased density of ORFs points toward a functional requirement and we hypothesize that the increase in ORF density is a consequence of the specific loss of termination codons in these mitochondrial genes. In several instances, alternative open reading frames start with a non-conventional initiation codon. This is not unusual and has been observed previously in edited and non-edited mitochondrial genes from different trypanosome species (Table S2). Furthermore, mitochondrial alternative initiation codons also have been observed in other protozoan and non-protozoan systems [26] [27].

We have been able to identify one potential gRNA for each of the alternatively edited mRNAs described. Based on these findings it is likely that gRNAs specify the protein coding information in alternatively edited mRNAs and that the mechanism of RNA editing does not differ for mRNAs encoding conventional or alternative mitochondrial mRNAs; different gRNAs are simply used. The diversity of the minicircle coding potential has always been a puzzling phenomenon as the number of minicircle encoded gRNA genes far exceeds the diversity needed for editing of the conventional mitochondrial mRNAs [15], [28]. Here we show that some of these gRNAs are engaged in alternative editing of mRNAs, leading to novel ORFs.

It is unknown what controls the selection of gRNAs at junction regions when gRNAs for both conventional and alternative editing can basepair with partially edited mRNA. Currently, the database of gRNA coding sequences in *T. brucei* is incomplete and with the addition of information from ongoing sequencing projects of small RNAs we anticipate the identification of all gRNAs necessary for the editing of mRNAs for conventional and alternative mitochondrial proteins (Ochsenreiter and Hajduk, unpublished results). Bioinformatic comparison of alternative and conventional gRNAs might reveal differences in primary or secondary structure and lead to an understanding of how selection of gRNAs for conventional or alternative editing is controlled.

## Materials and Methods

### Trypanosomes and RNA isolation

Pleomorphic long slender bloodstream form *T. brucei* (TREU 667) were harvested from infected rats at day three of infection at a parasitemia of 1–2×10^8^ cells/ml. Cells were lysed and mitochondria were purified as described by Harris *et al* (1990). RNA was isolated from mitochondria using the TriPure Isolation reagent (Boehringer Mannheim, Mannheim, Germany).

### Cloning and sequencing

Minicircles and gRNAs were cloned and sequenced as described previously [15]. Mitochondrial cDNA libraries from bloodstream *T. brucei* mitochondrial mRNAs were made from 5 µg of mitochondrial RNA using the Creator SMART cDNA library construction kit (Clontech, Palo Alto, USA). First and second strand cDNA were synthesized using poly-dT oligonucleotides and the SMART strand switching technology. In a second step we amplified (24 cycles) the cDNAs using oligonucleotides specific for the 5′ UTR of ND7, ND8, ND9, COXIII and A6 as well as the manufacturers 3′ oligonucleotide (creator smart 3′). Multiple bands can be seen on the agarose gels (Figure S1) they reflect the differentially edited mRNA populations from one gene sequence. Sequencing was done using the universal primer sequences (M13F and M13R) in the vector and only high quality sequences (Phred Q >20) were used for the analysis. For ND7 a second primer set was derived to create sufficient high quality sequence (Table S3).

### Bioinformatics

All bioinformatics analysis was carried out using the EMBOSS software package and our previously published database KISS [15], [29]. Potential gRNAs were predicted using WUBLAST with a modified matrix to allow for G:U base pairings. The prediction of open reading frames was done with GETORF from the EMBOSS package using the protozoan mitochondrial genetic code EGC.4 from NCBI [30].

## Supporting Information

Figure S1 (0.06 MB PDF)

Table S1 (0.04 MB DOC)

Table S2 (0.06 MB DOC)

Table S3 (0.03 MB DOC)
