# Supplementary material for: Alternative mRNA Editing in Trypanosomes Is Extensive and May Contribute to Mitochondrial Protein Diversity
Source: PLoS One. 2008 Feb 13;3(2):e1566. doi: 10.1371/journal.pone.0001566 (PMC2215773; doi:10.1371/journal.pone.0001566)
Supplement: Figure S1 — (0.06 MB PDF) [file pone.0001566.s001.pdf]

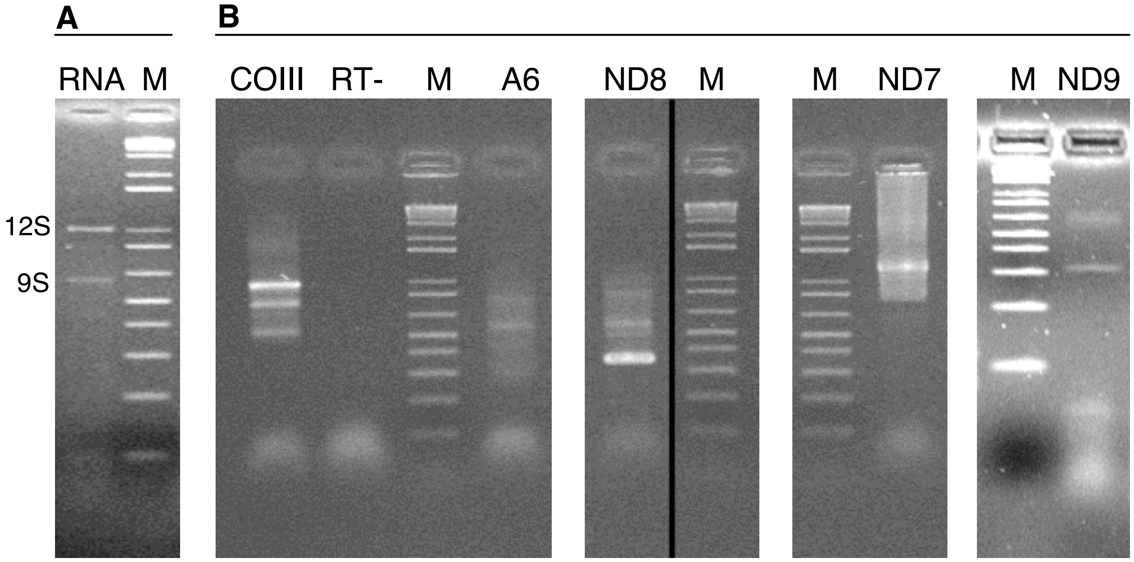

Figure S1: Gel electrophoresis of RNA and cDNA samples used in this study. **A)** Methylmercury agarose gel with mitochondrial RNA from purified mitochondria showed the two mitochondrial rRNAs (12S and 12S). **B)** cDNA samples after amplification with gene specific primers. Note the multiple bands (smear) from each cDNA. This reflects the diverse editing patterns of the individual mRNAs. RT- shows a representative PCR product from the cDNA reaction without reverse transcriptase added. M, marker bands from the bottom up are 100, 200 300, 400, 500 (bright band), 650, 850 1000 base pairs.
