# Supplementary material for: Alternative mRNA Editing in Trypanosomes Is Extensive and May Contribute to Mitochondrial Protein Diversity
Source: PLoS One. 2008 Feb 13;3(2):e1566. doi: 10.1371/journal.pone.0001566 (PMC2215773; doi:10.1371/journal.pone.0001566)
Supplement: Supplementary file 2 [file pone.0001566.s002.doc]

Table S1: Coding potential of the pre-edited mitochondrial genes in *T. brucei*

Gene	# ORFs in pre-edited sequence (size in amino acids) [1]	Length of pre-edited message (nucleotides)	# ORFs in pre-edited sequence on opposite strand of the gene (size in amino acids)
ND8	3 (107, 96, 110)	361	2 (98, 108)
ND9	1 (38)	322	3 (90, 81,76)
ND7	0	783	2 (96, 59)
COXIII	3 (65, 120, 153)	463	3 (62, 126, 131)
A6	2 (79, 97)	401	1 (79)
CR3	2 (35, 38)	164	2 (52, 36)
CR4	2 (63, 79)	283	2 (76, 77)
ND3	1 (77)	268	3 (58, 59, 79)
RPS12	1 (61)	221	2 (62, 71)

[1] Initiation within the first 60 nts, orf at least 150 nts (except for CR3 and ND9, here the limit was set to 100 nts)
