# Supplementary material for: Alternative mRNA Editing in Trypanosomes Is Extensive and May Contribute to Mitochondrial Protein Diversity
Source: PLoS One. 2008 Feb 13;3(2):e1566. doi: 10.1371/journal.pone.0001566 (PMC2215773; doi:10.1371/journal.pone.0001566)
Supplement: Supplementary file 3 [file pone.0001566.s003.doc]

Table S2: Alternative inititation codons in trypanosomes

Maxicircle Gene[1]	Editing[2]	Developmental Regulation[3]	Alternative Transcripts[4]	Alternative Open Reading Frames (ORFs)[5]	Initiation Codon[6]	Reference
**Complex I**						
ND1	Not edited	Unknown	Not known		UUG	
ND3	Insertion: 210<br>Deletion: 13	BF and PF	IPF and BS 3' region Alt. Ed.	Multiple C-termini	UUG	Read et al. 1994
ND8	Insertion: 259<br>Deletion: 46	BF-Abundant	BF 5' region pre-edited	2 alternative ORFs	AUG (alt ORF1 UUG, ORF2 GUG)	Souza et al., 1992<br>This manuscript
ND9	Insertion: 345<br>Deletion: 20	BF-Abundant	BF 5' region Alt. Ed.	1 alternative ORF	AUG (alt. ORF GUG or UUG)	Souza et al., 1993<br>This manuscript
**Complex III**						
Cyb	Insertion: 39<br>Deletion: none	PF-Abundant			AUU or UUG	Feagen et al., 1987
COIII	Insertion: 547<br>Deletion: 41	BF and PF	AEP-1	3 alternative ORFs	AUG or UUG	Feagin et al., 1988<br>Ochsenreiter & Hajduk 2006
**Unknown Function**						
MURF1	Not edited	Unknown			UUG	
CR3	Insertion: 148<br>Deletion: 13	BF-abundant	Not known		AUA or UUG	Stuart et al., 1997
CR4	Insertion: 325<br>Deletion: 40	BF-abundant	In PF the 5' region Alt. Ed.	2 alternative ORFs	AUG (alt. ORF UUG)	Corell et al., 1994

[1]Abbreviations: ND, NADH ubiquinone oxidoreductase subunits 1, 3, 4, 5, 7, 8, 9; CYb, apocytochrome b; CO, cytochrome oxidase subunit III; MURF, maxicircle unidentified reading frame; CR, G- versus C-strand biased gene subunits 3 and 4;

[2]Number of uridines inserted or deleted for each mRNA

[3]BF, bloodstream form, and PF, insect (procyclic) forms of *T. brucei*

[4]Alternatively edited (alt. ed.) mRNAs that differ from the predicted bona fide mRNA

[5]Alternatively edited mRNAs can have unique open reading frames (ORFs)

[6]Canonical and alternative initiation codons that result in the longest ORF are shown

References:

1. Corell RA, Myler P, Stuart K (1994) Trypanosoma brucei mitochondrial CR4 gene encodes an extensively edited mRNA with completely edited sequence only in bloodstream forms. Mol Biochem Parasitol 64: 65-74.
2. Feagin JE, Jasmer DP, Stuart K (1987) Developmentally regulated addition of nucleotides within apocytochrome b transcripts in Trypanosoma brucei. Cell 49: 337-345.
3. Feagin JE, Stuart K (1988) Developmental aspects of uridine addition within mitochondrial transcripts of Trypanosoma brucei. Mol Cell Biol 8: 1259-1265.
4. Ochsenreiter T, Hajduk SL (2006) Alternative editing of cytochrome c oxidase III mRNA in trypanosome mitochondria generates protein diversity. EMBO Rep 7: 1128-1133.
5. Read LK, Goringer HU, Stuart K (1994) Assembly of mitochondrial ribonucleoprotein complexes involves specific guide RNA (gRNA)-binding proteins and gRNA domains but does not require preedited mRNA. Mol Cell Biol 14: 2629-2639.
6. Souza AE, Myler PJ, Stuart K (1992) Maxicircle CR1 transcripts of Trypanosoma brucei are edited and developmentally regulated and encode a putative iron-sulfur protein homologous to an NADH dehydrogenase subunit. Mol Cell Biol 12: 2100-2107.
7. Souza AE, Shu HH, Read LK, Myler PJ, Stuart KD (1993) Extensive editing of CR2 maxicircle transcripts of Trypanosoma brucei predicts a protein with homology to a subunit of NADH dehydrogenase. Mol Cell Biol 13: 6832-6840.
8. Stuart K, Allen TE, Kable ML, Lawson S (1997) Kinetoplastid RNA editing: complexes and catalysts. Curr Opin Chem Biol 1: 340-346.
