# Supplementary material for: Alternative mRNA Editing in Trypanosomes Is Extensive and May Contribute to Mitochondrial Protein Diversity
Source: PLoS One. 2008 Feb 13;3(2):e1566. doi: 10.1371/journal.pone.0001566 (PMC2215773; doi:10.1371/journal.pone.0001566)
Supplement: Supplementary file 4 [file pone.0001566.s004.doc]

Table S3: Oligonucleotides used in this study

Primer target	Primer sequence	Annealing temperature °C
M13F	TCCCAGTCACGACGTCGT	54
M13R	CAGGAAACAGCTATGAC	54
COIII 5’	ATTGAGGATTGTTTAAAATT	54
A6 5’	TAGTATTTTGATATTATTAAAGT	54
ND7 5’	AAACATGACTACATGATAAGTA	54
ND7 211F	GAAGACATTGTTCTACACTTTT	48
ND7 839R	ATCAACAAACTCATACGCATAT	48
ND8 5’	TTAATAATTTTAAGTTTTGGTTG	54
ND9 5’	TATCAACTTAATTTTTTTTATAAAC	48
Creator SMART 3’	ATTCTAGAGGCCGAGGCGGCCGACATG-d(T)$_{30}$N–1N	42 (1st strand synthesis)
Creator SMART 5’	AAGCAGTGGTATCAACGCAGAGTGGCCATTACGGCCGGG	42 (1st strand synthesis)

N = A, G, C, or T; N–1 = A, G, or C
